# Racial disparities in central line-associated bloodstream infections: the impact of the COVID-19 pandemic

**DOI:** 10.1017/ice.2024.147

**Published:** 2024-11

**Authors:** Michael E. DeWitt, Mindy M. Sampson, Shelley Kester, Emily MacNeill, Catherine Passaretti

**Affiliations:** 1 Section on Infectious Diseases, Department of Internal Medicine, Wake Forest University School of Medicine, Winston-Salem, NC, USA; 2 Department of Biology, Wake Forest University, Winston-Salem, NC, USA; 3 Division of Infectious Diseases & Geographic Medicine, Department of Medicine, Stanford University, Stanford, CA, USA; 4 Department of Infection Prevention, Atrium Health, Charlotte, NC, USA; 5 Emergency Medicine, Atrium Health, Charlotte, NC, USA

## Abstract

This retrospective cohort study analyzed differences in rates of central line-associated bloodstream infections (CLABSI) in Black and White inpatients across 11 southeastern US hospitals from 2019 to 2021. Results showed higher CLABSI rates in Black patients during the coronavirus disease 2019 (COVID-19) pandemic, even after adjustment for COVID-19 infection and clinical factors.

## Introduction

Racial and ethnic disparities in healthcare outcomes are well-documented and multifaceted, involving the patient, provider, and systemic racism in the healthcare system and society^1^. These complex factors can lead to differences in underlying medical conditions, healthcare access, and care-seeking delays, which contribute to inequities^2^. The coronavirus disease 2019 (COVID-19) pandemic exacerbated disparities, disproportionately affecting underserved populations and communities of color^3^. Concurrently, hospitals saw worsening in several healthcare quality metrics including central line-associated bloodstream infections (CLABSI)^4,5^. During periods of heightened stress such as the COVID-19 pandemic, time pressure, higher patient loads, and provider burnout have been linked to greater implicit racial biases, negatively impacting health care^6–8^.

Although several studies suggest racial disparities in CLABSIs^9^, data remains limited. This study investigates race and CLABSI before and during the COVID-19 pandemic.

## Methods

### Study design and setting

We conducted a retrospective cohort study of non-Hispanic Black and non-Hispanic White adult patients with central lines admitted between January 1, 2019, to December 31, 2021, to 11 hospitals in North Carolina. Due to small group sizes (less than 8% of the total population), patients from other races and ethnicities were excluded from the analysis. CLABSIs were identified by trained infection preventionists using standard definitions^10^ and linked to patient encounters. Demographics, address, medical comorbidities, central line characteristics, time to CLABSI event, hospital length of stay, and death within 30 days of CLABSI event were collated from the electronic health record. This study was approved by the Wake Forest University School of Medicine Institutional Review Board.

### Definitions and data sources

Time to CLABSI was calculated from the date of line insertion (or first access) to the date of the CLABSI event. Race was determined by the patient’s report as documented in the medical record at the time of registration. Before the pandemic was defined as January 1, 2019, through February 29, 2020, and during the pandemic as March 1, 2020, through March 31, 2021. Patients with COVID-19 were patients with at least one severe acute respiratory coronavirus virus 2 (SARS-CoV-2) positive test during the hospitalization.

### Statistical analysis

Clinical characteristics of patients with central lines and CLABSIs were compared between Black and White patients before and during the pandemic using χ^2^, Fisher’s exact, and Wilcoxon rank sum tests to assess differences between groups. Variables were reported as medians with interquartile range or as frequencies. *P*-values <.05 were considered significant.

The primary outcome was the first CLABSI event during an encounter with patients censored at the time of line removal, death, or discharge. CLABSI rates per 1000 central line days and central line utilization rates per 10,000 patient days were compared between Black and White patients before and during the pandemic. Patient days in the utilization calculations included all patients admitted.

Univariate and multivariate Cox proportional hazard models assessed the risk of a CLABSI by race, stratified by pandemic period. Baseline clinical differences between White and Black patients were imputed into a causal directed acyclic graphic (DAG) based on the relationship between features using clinical knowledge. Using the DAG, COVID-19 status, total parenteral nutrition (TPN), dialysis, BMI (body mass index), Charlson Comorbidity Index, diabetes mellitus, and site of care were selected as potential confounders and mediators of the effect of race on the likelihood of CLABSI. These variables were added to our adjusted model.

Analyses were conducted in R version 4.1.3.

## Results

### Clinical characteristics

A total of 42,888 patients met the inclusion criteria. White patients comprised 65.1% of our population with central lines. During the pandemic, there was an increase in central line patients with Medicaid (*P* < .001) and diabetes (*P* < .001) and a decrease in those with cancer (*P* < .001) or transplant (*P* = .001). There was also a shift in line types, with more dialysis lines and fewer ports (*P* < .001) (Table 1).

**Table 1.** Demographic and clinical characteristics of patients with a central line

			Stratified by period of time					Stratified by race				
	All patients n = 42,888		Before the COVID-19 pandemic^a^ n = 17,842		During the COVID-19 pandemic^b^ n = 25,046		*p*-value^c^	White n = 27,958		Black n = 14,930		*p*-value^c^
**Demographics**												
Age (years), Median (IQR)^d^	61	(50–71)	62	(50–71)	61	(50–71)	.89	63	(52–72)	58	(46–68)	<.01
Sex, n (%)							.02					<.01
Female	21,269	(49.6)	8,971	(50.3)	12,298	(49.1)		13,485	(48.2)	7,784	(52.1)	
Race, n (%)							.02					
Non-Hispanic White	27,958	(65.7)	11,743	(65.8)	16,215	(64.7)						
Non-Hispanic Black	14,930	(35.3)	6,099	(34.2)	8,831	(35.3)						
**Social determinants, n (%)**												
Insurance							<.01					<.01
Medicaid	6,603	(15.4)	2,613	(14.6)	3,990	(15.9)		3,210	(11.5)	3,393	(22.7)	
Medicare	22,027	(51.4)	9,329	(52.3)	12,698	(50.7)		14,199	(50.8)	7,828	(52.4)	
Private/commercial	12,007	(28.0)	5,015	(28.1)	6,992	(27.9)		9,103	(32.6)	2,904	(19.5)	
Self-pay	2,251	(5.2)	885	(5.0)	1,366	(5.5)		1,446	(5.2)	805	(5.4)	
Rurality^e^							.50					<.01
Urban	34,695	(80.9)	14,412	(80.8)	20,283	(81.0)		21,725	(77.7)	12,970	(86.9)	
Large rural city/town	7,173	(16.7)	3,017	(16.9)	4,156	(16.6)		5,458	(19.5)	1,715	(11.5)	
Small rural town	603	(1.4)	235	(1.3)	368	(1.5)		484	(1.7)	119	(0.8)	
Isolated small rural town	375	(0.9)	156	(0.9)	219	(0.9)		264	(0.9)	111	(0.7)	
Unknown	42	(0.1)	22	(0.1)	20	(0.1)		27	(0.1)	15	(0.1)	
**Clinical characteristics, n (%)**												
COVID-19	2,484	(5.8)	1	(0.0)	2,483	(100.0)		1,557	(5.6)	927	(6.2)	<.01
Diabetes	23,991	(55.9)	9,790	(54.9)	14,201	(56.7)	<.01	15,074	(53.9)	8,917	(59.7)	<.01
Dialysis	7,352	(17.1)	3,054	(17.1)	4,298	(17.2)	.91	2,447	(8.8)	4,905	(32.9)	<.01
Total parenteral nutrition	3,094	(7.2)	1,273	(7.1)	1,821	(7.3)	.59	2,195	(7.9)	899	(6.0)	<.01
Transplant	2,870	(6.7)	1,282	(7.2)	1,588	(6.3)	<.01	1,644	(5.9)	1,226	(8.2)	<.01
Cancer	15,670	(36.5)	6,857	(38.4)	8,813	(35.2)	<.01	11,048	(39.5)	4,622	(31.0)	<.01
Body mass index, median (IQR)^d^	28	(24–33)	28	(23–33)	28	(24–34)	<.01	28	(24–33)	28	(23–34)	.45
Charlson Comorbidity Index^f^, median (IQR)^d^	7	(4–10)	7	(4–10)	7	(4–10)	.04	7	(4–10)	7	(4–11)	<.01
Length of stay (days), median (IQR)^d^	7	(4–13)	7	(4–12)	7	(4–14)	<.01	7	(4–13)	7	(4–13)	.49
**Central line characteristics**												
Line days, Median (IQR)^d^	3	(2–6)	3	(2–6)	3	(2–7)	<.01	3	(2–6)	3	(1–6)	.54
Type of central line, n (%)							<.01					<.01
Peripherally inserted central venous catheter	11,429	(26.6)	4,799	(26.9)	6,630	(26.5)		8,317	(29.7)	3,112	(20.8)	
Dialysis	4,640	(10.8)	1,767	(9.9)	2,873	(11.5)		1,838	(6.6)	2,802	(18.8)	
Nontunneled central line	10,397	(24.2)	4,281	(24.0)	6,116	(24.4)		7,397	(26.5)	3,000	(20.1)	
Implanted port	9,498	(22.1)	4,271	(23.9)	5,227	(20.9)		6,417	(23.0)	3,081	(20.6)	
Tunneled central line	1,150	(2.7)	403	(2.3)	747	(3.0)		724	(2.6)	426	(2.9)	
Multiple	299	(0.7)	69	(0.4)	230	(0.9)		189	(0.7)	110	(0.7)	
Unknown	5,475	(12.8)	2,252	(12.6)	3,223	(12.9)		3,076	(11.0)	2,399	(16.1)	
**Pandemic period, n (%)**												
Before the COVID-19 Pandemic^a^	17,842	(42)	N/A		N/A			11,743	(42)	6,099	(41)	.02
During the COVID-19 Pandemic^b^	25,046	(58)	N/A		N/A			16,215	(58)	8,831	(59)	

a January 1, 2019–February 29, 2020.

b March 1, 2020–December 31, 2021.

c Wilcoxon rank sum test; Pearson’s χ^2^ test.

d IQR, interquartile range.

e Rurality categorization was assigned using the US Census’s Rural-Urban Commuting Area Codes (RUCA) consolidated using the Categorization E approach defined by the University of Washington’s Rural Health Research Center guidelines (https://depts.washington.edu/uwruca/ruca-uses.php).

f Age adjusted.

Black patients with a central line were younger (*P* < .001), more likely to be female (*P* < .001), have Medicaid (*P* < .001), and come from an urban area compared to White patients. Black patients had higher rates of comorbidities including COVID-19 (*P* = .006), diabetes (*P* < .001), dialysis dependence (*P* < .001), and transplant history (*P* < .001). White patients were more likely to be on TPN (*P* < .001) or have a cancer diagnosis (P < .001). Central line types also varied by race with White patients more likely to have peripherally inserted central venous catheters or nontunneled catheters, and Black patients more likely to have dialysis catheters (*P* < .001) (Table 1).

### CLABSI rate and central line utilization

During the 3-year period, 274 CLABSI were reported in 247,414 central line days (1.11 CLABSI per 1000 central days) and 2,005,552 patient days (central line utilization rate 1233 per 10,000 patient days). CLABSI rates and central line utilization were higher during the pandemic, especially in patients with COVID-19.

**Figure 1.**
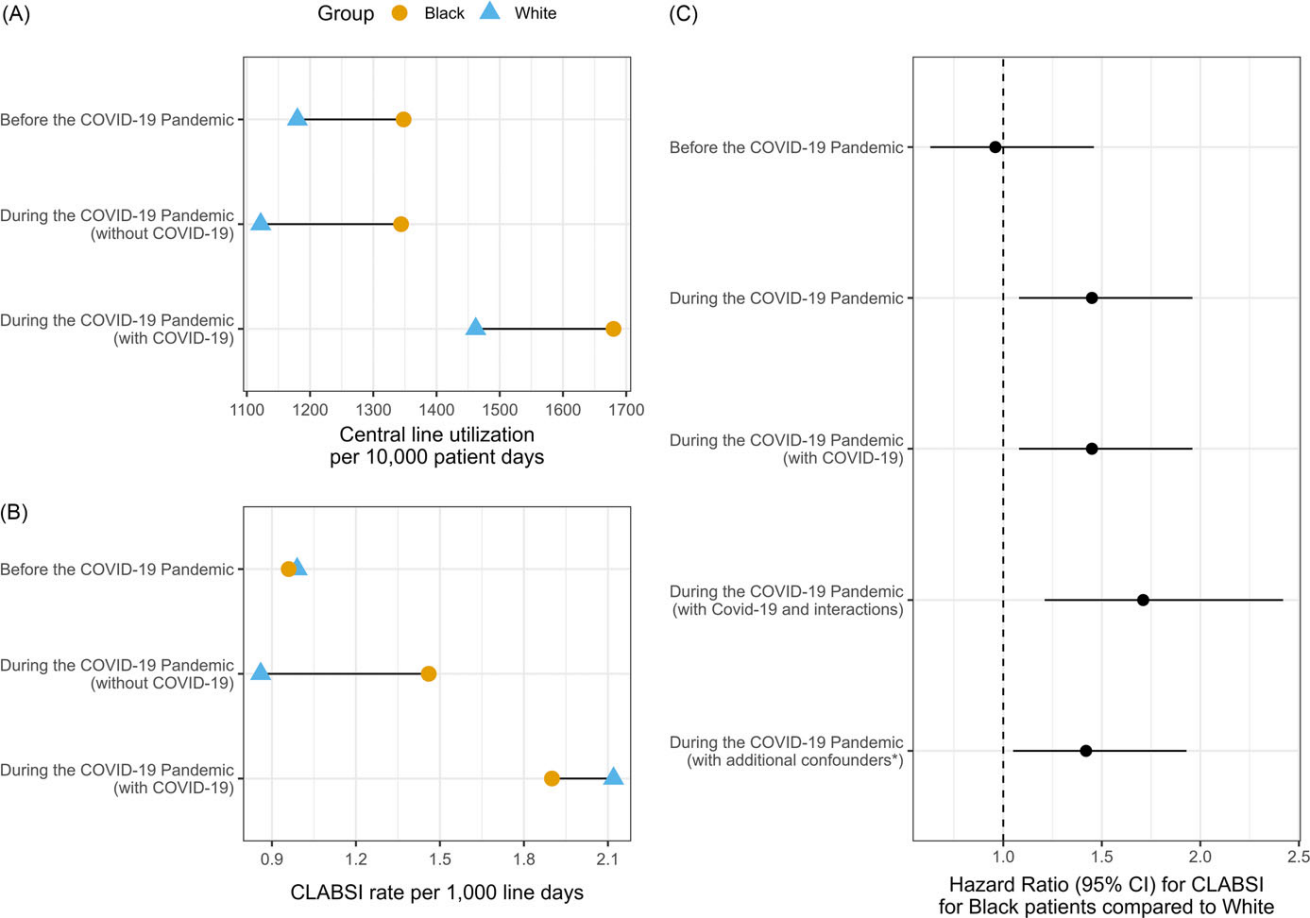
Central line utilization and CLABSI rate prior to and during the COVID-19 pandemic. Panel (A) displays central line utilization rates per 10,000 patient days prior to and during the COVID-19 pandemic stratified by race and whether COVID-19 was detected during the hospitalization where the central line was in place. In all periods and regardless of COVID-19 status, Black patients (●) had significantly higher central line utilization rates than White patients (▴). Panel (B) displays central line-associated bloodstream infection rates per 1000 central line days stratified by race and COVID-19-positive status. Prior to the pandemic and during the pandemic for patients with COVID-19 detected, rates of CLABSI were similar. CLABSI rates in Black patients (●) without COVID-19 detected during the pandemic were significantly higher than White patients (▴) without COVID-19 detected. Panel (C) displays the hazard ratios for a CLABSI for Black patients compared to White patients during the different pandemic periods and considering different confounders in multivariable Cox regression models. Black patients (●) had higher hazards of CLABSI during the pandemic period even when adjusting for COVID-19 status, total parenteral nutrition, dialysis, body mass index, Charlson Comorbidity Index, diabetes, and site of care. COVID-19, coronavirus disease 2019; CLABSI, central line-associated bloodstream infections.

CLABSI rates in Black and White patients were similar before the pandemic (0.96 vs 0.99 per 1000 central line days, *P* = .8) and for patients with COVID-19 during the pandemic (1.9 vs 2.1 per 1000 central line days, *P* = .9). However, Black patients without COVID-19 during the pandemic had significantly higher CLABSI rates (1.5 vs 0.9 per 1000 central line days, *P* = .008). Central line utilization was higher (*P* < .001) in Black patients across all periods (Figure 1).

### Risk factors for CLABSI

Univariate analysis showed no significant racial differences in CLABSI risk before the pandemic. During the pandemic, Black race (*P* = .009) and COVID-19 infection (*P* = .003) were associated with a significantly higher risk of CLABSI, while having a cancer diagnosis was associated with a lower risk (*P* = .01) (Supplemental Figure 1). Multivariable analysis showed that Black patients were 1.44 times more likely to develop a CLABSI than White patients during the pandemic even after adjusting for TPN, dialysis, BMI, Charlson Comorbidity Index, diabetes, site of care, and COVID-19 infection (*P* = .02) (Figure 1).

### Outcomes by race in patients with CLABSI

30-day all-cause mortality for patients with CLABSI was 23%, with no significant differences between Black and White patients (*P* = .8). Lengths of stay were also similar (median 28 days, *P* = .8) (Supplemental Table 1).

## Discussion

During the pandemic, we observed increased CLABSI rates, with a greater increase in Black patients compared to White patients, a disparity that was not present before the pandemic. We observed changes in clinical characteristics that may influence CLABSI risk. Black patients had higher rates of dialysis dependence, diabetes, and central line utilization. Shifts in central line patient characteristics during the pandemic reflect changes in healthcare-seeking behavior and the impact of patients admitted with severe COVID-19 infection. During this period, there were fewer patients with a central line with cancer or transplants. Additionally, we saw changes in central line types with more dialysis catheters and fewer ports, indicating a shift in the patient population.

The COVID-19 pandemic’s differential impact on communities of color might suggest that disparities in CLABSI risk were driven by increased COVID-19 admissions among Black patients. However, Black patients with central lines were 1.4 times more likely to develop a CLABSI than White patients even after adjusting for comorbidities including COVID-19, suggesting Black patients may be experiencing inequitable care.

Limitations of our study include potential information bias due to reliance on electronic health record data. Misclassification or documentation gaps could have occurred in race identification, comorbidities, and central line characteristics. Reliance on lab results for COVID-19 results could underrepresent this diagnosis. Results may not be generalizable to other settings.

Further research is essential to understand if inequities in clinical care contribute to disparities in healthcare-associated infections, particularly during increased healthcare stressors.

## Supporting information

DeWitt et al. supplementary material 1 DeWitt et al. supplementary material

DeWitt et al. supplementary material 2 DeWitt et al. supplementary material

## Supplementary material

To view supplementary material for this article, please visit https://doi.org/10.1017/ice.2024.147
