# Supplementary material for: Racial disparities in central line-associated bloodstream infections: the impact of the COVID-19 pandemic
Source: Infect Control Hosp Epidemiol. 2024 Sep 26;45(11):1350–4. doi: 10.1017/ice.2024.147 (PMC11663464; doi:10.1017/ice.2024.147)
Supplement: DeWitt et al. supplementary material 1 — DeWitt et al. supplementary material [file S0899823X24001478sup001.docx]

Supplemental Table 1: Characteristics of Patients with a Central Line Associated Blood Stream Infection

| **Demographics, n (%)** | **Black**, N = 111 | **White**, N = 164 | **p-value^a^** |
| --- | --- | --- | --- |
| Age (years), Median (IQR) | 58 (46 – 65) | 59 (49 – 69) | 0.086 |
| Sex |  |  | 0.078 |
| Female | 58 (52) | 68 (41) |  |
| **Social Determinants, n (%)** |  |  |  |
| Insurance |  |  | 0.002 |
| Medicaid | 30 (27) | 23 (14) |  |
| Medicare | 57 (51) | 72 (44) |  |
| Private/Commercial | 21 (19) | 57 (35) |  |
| Self-pay | 3 (2.7) | 12 (7.3) |  |
| Rurality |  |  | 0.058 |
| Urban | 93 (84) | 116 (71) |  |
| Large Rural City/ Town | 16 (14) | 42 (26) |  |
| Small Rural Town | 1 (0.9) | 4 (2.5) |  |
| Isolated Small Rural Town | 1 (0.9) | 1 (0.6) |  |
| Unknown | 0 | 1 |  |
| **Clinical Characteristics, n (%)** |  |  |  |
| Diabetes | 68 (61) | 94 (57) | 0.51 |
| Dialysis | 29 (26) | 15 (9.1) | <0.001 |
| Total Parenteral Nutrition | 28 (25) | 52 (32) | 0.25 |
| Cancer | 31 (28) | 56 (34) | 0.28 |
| Transplant | 11 (9.9) | 15 (9.1) | 0.83 |
| Death | 25 (23) | 37 (23) | >0.99 |
| COVID-19 | 16 (14) | 32 (20) | 0.27 |
| Charlson Comorbidity Index^b^, Median (IQR) | 7.0 (4.0 – 9.0) | 6.0 (3.0 – 9.0) | 0.56 |
| Length of stay (days), Median (IQR) | 28 (15 – 46) | 28 (14 – 48) | 0.99 |
| **Central Line Characteristics** |  |  |  |
| Line days, Median (IQR) | 16 (9 – 29) | 18 (9 – 32) | 0.56 |
| Type of central line, n (%) |  |  | 0.019 |
| PICC | 29 (28) | 78 (48) |  |
| Dialysis | 21 (20) | 25 (15) |  |
| Non-tunneled CL | 28 (27) | 27 (17) |  |
| Implanted port | 13 (12) | 15 (9.3) |  |
| Tunneled CL | 4 (3.8) | 9 (5.6) |  |
| Multiple | 10 (9.5) | 8 (4.9) |  |
| Unknown | 6 | 2 |  |
| a Wilcoxon rank sum test; Pearson's Chi-squared test  b Age adjusted | | | |
