# Supplementary figures and images for: Racial disparities in central line-associated bloodstream infections: the impact of the COVID-19 pandemic
Source: Infect Control Hosp Epidemiol. 2024 Sep 26;45(11):1350–4. doi: 10.1017/ice.2024.147 (PMC11663464; doi:10.1017/ice.2024.147)

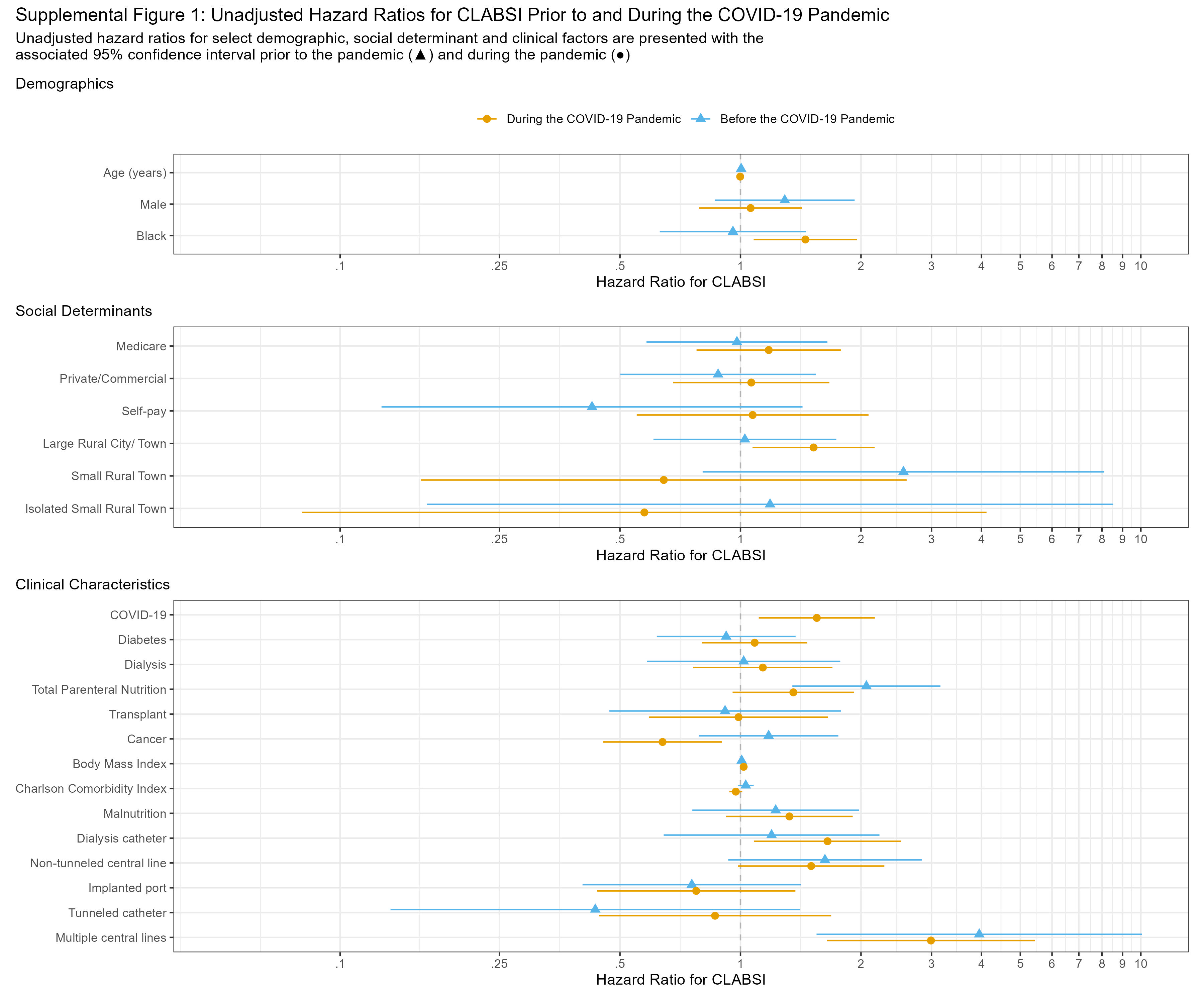

Supplement: DeWitt et al. supplementary material 2 — DeWitt et al. supplementary material [file S0899823X24001478sup002.zip › supplemental-figure-1.jpg]
